# Data on the first endurance activity of a Brushless DC motor for aerospace applications

**DOI:** 10.1016/j.dib.2020.105153

**Published:** 2020-01-22

**Authors:** Mirko Mazzoleni, Matteo Scandella, Fabio Previdi, Giulio Pispola

**Affiliations:** a University of Bergamo, Department of Management, Information and Production Engineering, Via Marconi 5, 24044, Dalmine, BG, Italy; b UmbraGroup S.p.A., Via Baldaccini 1, 06034, Foligno, PG, Italy

**Keywords:** Electro-mechanical actuators, Fault diagnosis, Condition monitoring, Predictive maintenance, Aerospace

## Abstract

This article describes the data acquired during the first test activity carried out in the Reliable Electromechanical actuator for PRImary SurfacE with health monitoring (REPRISE) H2020 project. The data consist of a set of measures from an Electro-Mechanical Actuator (EMA) employed in small aircrafts, such as phase currents, positions, temperature and loads. A test bench was developed to perform endurance sessions in various loads and working conditions. Specifically, two datasets are provided: (i) measurements used to monitor the EMA degradation through time; (ii) measurements that characterize the EMA closed-loop dynamic behaviour in healthy condition. The data are helpful to develop and test system identification methods and condition monitoring approaches.

**Table:** Specifications Table

Subject	Engineering
Specific subject area	Control and Systems Engineering
Type of data	Table Chart Figure
How data were acquired	Data acquired through two sets of National Instruments hardware and LabVIEW software.
Data format	Raw and analyzed
Parameters for data collection	Phase currents data were acquired at 4800 Hz, while positions and load data were acquired at 100 Hz. Position variables were acquired also at 500 Hz for synchronization purposes.
Description of data collection	The EMA was actuated with sinusoidal input profiles at different frequencies. Measured tests for development of health monitoring algorithms were performed at 300 N load, between test with 800 N load to stress the actuator. In this case, the EMA transmission was configured with normal, poor and no lubricant. Measured tests for computation of the Bode diagram of the system were performed with 0 N load. In this case, the EMA was in healthy condition.
Data source location	Institution: Umbra Group, via Baldaccini 1 06034 City/Town/Region: Foligno (PG) Country: Italy
Data accessibility	Repository name: Mendeley Data Data identification number: https://doi.org/10.17632/m58bdhy2df.2 Direct URL to data: https://data.mendeley.com/datasets/m58bdhy2df/2
Related research article	M. Mazzoleni, F. Previdi, M. Scandella and G. Pispola, “Experimental Development of a Health Monitoring Method for Electro-Mechanical Actuators of Flight Control Primary Surfaces in More Electric Aircrafts,” in IEEE Access, vol. 7, pp. 153618–153634, 2019. doi: 10.1109/ACCESS.2019.2948781

**Table:** 

**Value of the Data** • The data are useful for developing and testing system identification, fault diagnosis and condition monitoring algorithms. They consist in a health monitoring dataset and a closed-loop dynamic behaviour dataset. The latter is particularly useful in the system identification use case. In particular, we observed a dynamic behaviour that depends on the amplitude of the sinusoidal input reference signal. Thus, techniques for Linear-Parametric-Variant (LVP) systems can be employed. Fault diagnosis and condition monitoring algorithms can be tested on the health monitoring dataset, where several key variables of an EMA are measured across a continuous endurance and monitoring activities. • The data can be beneficial both to researchers in the field of system identification and fault diagnosis, and to company engineers looking for a benchmark behaviour of a 1:1 EMA for aerospace applications. In fact, the proposed data contains experimental measurements (mainly phase currents and position) of a real flight EMA employed in small aircrafts. • The data can be further used for developing Residual Useful Life (RUL) estimation algorithms and to have an experimental case study with settings (loads, working conditions, number of EMA screw revolutions) already tested. • The additional value of the data lies in the long endurance activity performed in order to acquire them (about 1 month), that can benefit engineers looking for a ready-to-use comparison or benchmark of flight EMA degradation. Furthermore, the data come from a real 1:1 EMA application.

## 1 Data

In this article, we present the data acquired during the first endurance campaign of the Reliable Electromechanical actuator for PRImary SurfacE with health monitoring (REPRISE) project, a EU-H2020 funded project that aims at introducing Health Monitoring (HM) algorithms for Electro-Mechanical Actuators (EMAs) employed to control primary flight surfaces (aileron, elevator and rudder) in small aircrafts [1,2]. Two datasets are provided [3]:

1. Measurements used to monitor the EMA degradation through time;
2. Measurements that characterize the EMA closed-loop dynamic behaviour in healthy condition.

### 1.1 Health monitoring dataset

In order to develop the health monitoring algorithms, a test campaign has been carried out with the aim of performing degradation on the mechanical components of an EMA. The data were acquired from a 1:1 scale flight EMA, under different loads and operating conditions, see Ref. [1]. During the campaign, the EMA has undergone two different test types: endurance and monitoring tests. The aim of endurance tests was to stress the actuator to induce degradation in its mechanical components (mainly the ballscrew transmission). Between several endurance tests, monitoring tests were performed in order to acquire measurements from the system. The monitoring tests are the one that can be used to develop health monitoring algorithms, by assessing how the measured variables behaviour evolves from one monitoring test to another.

The considered health monitoring dataset consists in *11 monitoring tests*, acquired from *11 September 2017* to *12 October 2017*. We performed a monitoring test in the following dates: 11 September 2017, 18 September 2017, 21 September 2017, 25 September 2017, 02 October 2017 (2 tests), 03 October 2017, 04 October 2017, 09 October 2017, 11 October 2017, 12 October 2017. For each monitoring test, the main measured physical variables are reported in Table 1. Additional variables are present such that: a torquemeter, an additional external encoder and a diagnostic information. However, they are not included in Table 1 since their value is not reliable or it is not present in every test.

**Table 1:** Raw physical measurements acquired during monitoring tests.

#	Physical variable	Stored variable name	Sampling frequency (Hz)
1	Linear motor load reference (N)	load_cell_measure	100
2	Temperature inside the EMA box (°C)	temperature	100
3	Current supplied to the EMA from power supply (A)	linear_motor_supplied_current	100
4	Load cell measure (N)	load_cell_measure	100
5	EMA position reference from internal cRIO hardware (mm)	EMA_Position_reference_cRIO	100
6	EMA position measure from embedded LVDT sensor (mm)	EMA_LVDT_position	100
7	EMA position measure from external Renishaw absolute optical sensor (mm)	absolute_encoder_biss	100
8	EMA position reference from external cDAQ hardware (mm)	EMA_Position_reference_cDAQ	500
9	Current supplied to the linear motor from its drive (A/2)	linear_motor_drive_current	500
10	EMA phase current A (A)	phase_A	4800
11	EMA phase current B (A)	phase_B	4800
12	EMA phase current C (A)	phase_C	4800

Each experiment has its own measurement file: so, there are 11 files in the dataset, where each file is called data.mat. The files are converted into the MatLab data format (with extension.mat). The 12 raw variables of Table 1 are stored into each one of the MatLab files. Physical variables 1–7 are acquired by a National Instruments (NI) cRIO internal to the test bench at 100 Hz. Physical variables 8–9 are acquired from an external NI cDAQ hardware at 500 Hz. The phase currents are measured by an external cDAQ at 4800 Hz. For the first four tests, i.e. from 11 September 2017 to 25 September 2017, the *current sensor of the phase A did not work*. These data can be used for *sensor fault detection* or for a condition monitoring strategy that employs two phase current over the three available. As it is possible to observe from Table 1, the EMA reference position is acquired by each of the two acquisition hardware, in order to synchronize the measurements from different sources. More information about the measurements can be found in Refs. [1,2].

**Fig. 1:**
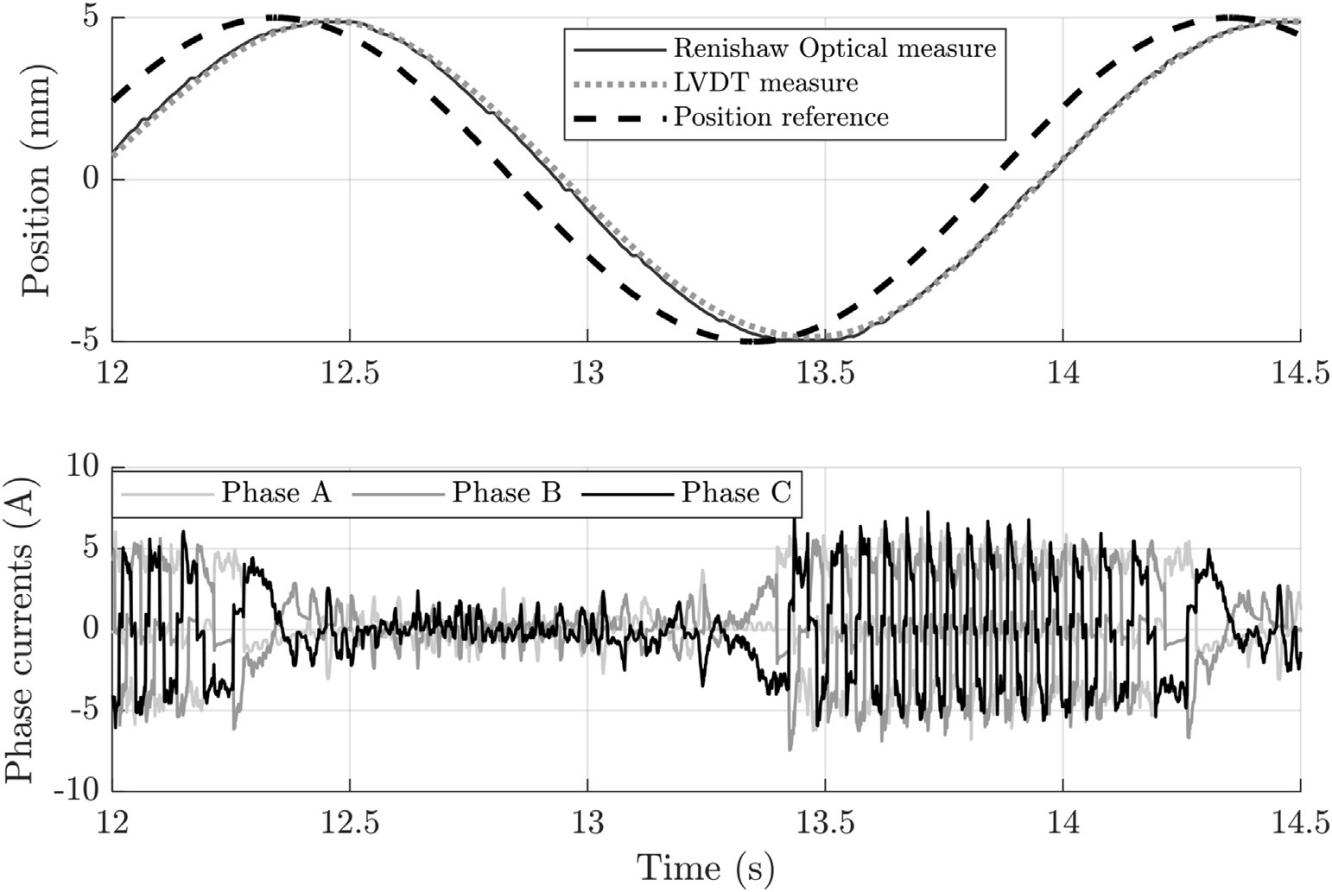
Example of position and phase current measurements from the EMA.

An example of measurements acquired from the EMA is shown in Fig. 1. The top plot shows the reference position input signal and the positions measured by the internal LVDT and external optical encoders. The bottom plot depicts the phase current measurements from the installed LEM sensors (see the Experimental setup section).

### 1.2 Closed-loop dynamic behaviour dataset

The second dataset contains data to compute the closed-loop Bode diagrams of the EMA in healthy condition, at four different input amplitudes. The dataset contains in *1 file,* called Bode.mat. This experimental session was performed 06 September 2017, and it consisted in actuating the EMA with different sinusoidal position profiles at various frequencies and amplitudes. Specifically, the following 60 frequencies were tested (in Hz): {0.1; 0.3; 0.5; from 0.6 to 3 with step of 0.05; 3.5; from 4 to 10 with step of 1}. The tested amplitudes were (in mm): {5; 10; 20; 30}. There are 49 periods of the input profile for each frequency and position. The first one was the discarded to remove transient and initialization effects. The reference and actual position variables were sampled at. 100 Hz.

**Fig. 2:**
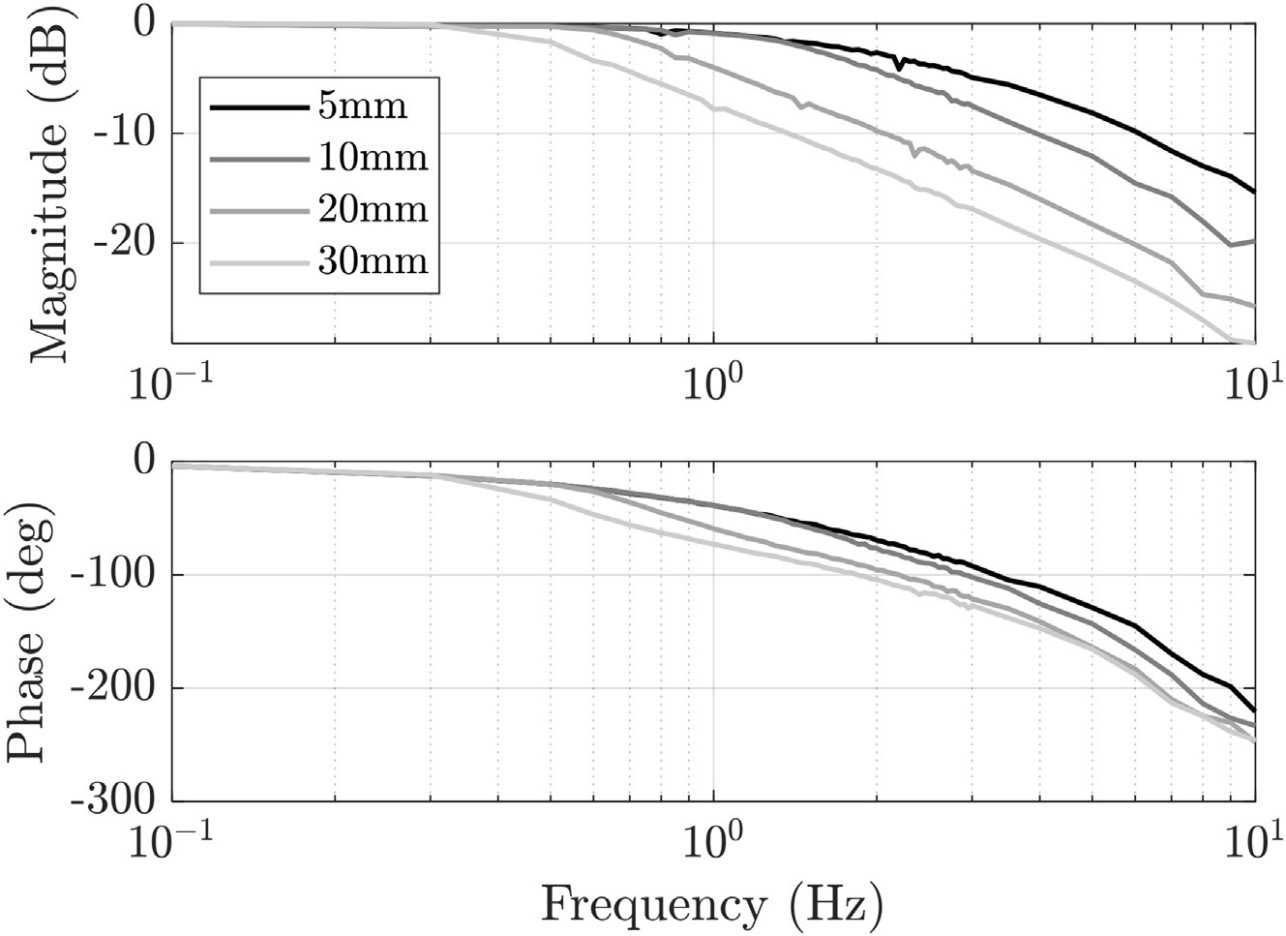
Position closed-loop Bode diagram of the EMA.

In healthy condition, the EMA presents a closed-loop varying dynamics that *depend on the amplitude* of the sinusoidal position reference. The diagrams of Fig. 2 show the experimental position closed-loop Bode diagrams of the EMA at different reference amplitudes, with 0 N load. The system can be considered a good benchmark for nonlinear system identification methods.

The variables included in the Bode.mat file are reported in Table 2. The dataset contains:

1. The raw input∖output data, already grouped to isolate each period of the sinusoidal input∖output profiles (variables tim_[source]). These variables contain a 4 × 60 cell array. Each element (one for each amplitude and frequency) is a 1 × 49 cell (one for each performed input∖output sine period) and each of these cells are 1 × 1001 arrays (the reference∖measured sinusoidal position measurements).
2. The second bin (the first bin is the constant component of the signal) of the Discrete Fourier Transform (DFT) of each input∖output sine period (variables dft_[source]). These variables contain a 4 × 60 cell array. Each element (one for each amplitude and frequency) is a 48x1 complex array (one for each considered input∖output sine period).
3. The computed Bode diagrams (variables Bode_[output_input]). These variables contain a 4x1 cell array. Each cell is a 60x1 complex array that defines the Bode diagram at a particular input amplitude.

**Table 2:** Variables stored for computing the EMA closed-loop Bode diagrams.

#	Meaning of the variable	Stored variable name
1	Bode diagram from speed reference to output LVDT sensor	Bode_LVDT_wRef
2	Bode diagram from position reference to output LVDT sensor	Bode_LVDT_xRef
3	Bode diagram from speed reference to output Ranishaw optical encoder	Bode_rani_wRef
4	Bode diagram from position reference to output Ranishaw optical encoder	Bode_rani_xRef
5	Second bin of the DFT of output position signals measured by the LVDT sensor	dft_LVDT
6	Second bin of the DFT of output position signals measured by the Ranishaw optical encoder	dft_rani
7	Second bin of the DFT of reference speed, computed from the position measured by the LVDT sensor	dft_wRef_LVDT
8	Second bin of the DFT of reference speed, computed from the position measured by the Ranishaw optical encoder	dft_wRef_rani
9	Second bin of the DFT of input reference position profile	dft_xRef
10	Periods of the position output measured by the LVDT sensor	tim_LVDT
11	Periods of the position output measured by the Ranishaw optical encoder	tim_rani
12	Periods of the reference speed, computed from the position measured by the LVDT sensor	tim_wRef_LVDT
13	Periods of the reference speed, computed from the position measured by the Ranishaw optical encoder	tim_wRef_rani
14	Periods of the input reference position profile	tim_xRef

## 2 Experimental Design, Materials, and Methods

This section briefly reviews the experimental activity carried out on the available EMA. For more information, see Ref. [1].

### 2.1 Experimental setup

The experimental setup consists in: (i) the EMA, (ii) the test bench employed for performing the tests and (iii) the acquisition setup. The EMA is a Brushless DC three-phases electrical motor with five poles and a ballscrew transmission, and it is closed-loop position controlled by a dedicated Electronic Control Unit (ECU). The EMA position is measured by a Linear Variable Displacement Transducer (LVDT) and three Hall sensors embedded in the actuator. The EMA operates at a maximum supply voltage of 28 Vdc. The ballscrew transmission consists of 8 circuits with 1 turn each and a specific number of balls per turn. Furthermore, an anti-rotation device that compensates for radial loads is present.

The test bench permits to actuate the EMA with sinusoidal positions, at different amplitudes and frequencies. It is also possible to define a position offset from where the EMA starts its motion. The EMA stroke is of ±30mm in the 0 offset position. The load that contrasts the EMA is generated by a Parker Ironcore R16-3A-HS linear motor that is closed-loop controlled using a load cell. An external Renishaw absolute optical encoder measures the position of the linear motor. A radial component equal to 17% of the axial load was applied to the EMA to increase its stress conditions and accelerate the degradation process.

The measured variables are acquired by two different hardware with National Instruments (NI) LabVIEW: a NI cRIO 9030 and a NI cDAQ 9188. The cRIO measures the variables 1–7 in Table 1, while variables 8–12 are measured by the cDAQ. The cRIO hardware is embedded in the test bench. While measuring the before-mentioned variables, it is also responsible for performing the control of the linear motor and generating the position reference for the EMA. The cDAQ performs only data acquisition: in particular, the three phase currents were measured by three LEM ATO-20-B333-D10 sensors (one for each phase).

### 2.2 Testing procedure

As briefly stated previously, two types of experiments were performed: endurance tests and monitoring tests. Endurance tests were performed with a load of 800 N (H1 condition). Monitoring tests were performed with a load of 300 N (H0 condition). An air-cooling system kept the EMA temperature at a fixed ambient level. Prior to the start of the tests, we removed the anti-rotation component to stress the actuator with higher forces.

Since the EMA can be commanded only by sinusoidal inputs, an experiment is completely defined by the frequency, amplitude and offset of the reference signal. A total of 10 frequencies for the reference position profile of the EMA are used (in Hz): {0.1; 0.3; 0.5; 0.8; 0.9; 1; 1.5; 2; 2.5; 4}. Then, each row of Table 3 (that denotes a single experiment) is repeated for each input frequency previously defined. Monitoring trials consist in measuring one repetition of Table 3. Endurance trials run Table 3 many times. A monitoring trial is performed roughly after ten Endurance trials.

**Table 3:** Position configurations in the experimental table.

Offset position (mm)	Amplitude position (mm)	Stroke range (mm)	No. of sinusoid periods
0	5	[-5, +5]	100
0	10	[-10, +10]	100
+10	5	[+5, +15]	100
+10	10	[0, +20]	100
−10	5	[-15, −5]	100
−10	10	[-20, 0]	100

The EMA has been tested in different lubricant conditions:

1. *Normal level lubricant*: this condition concerns the monitoring tests of 11 September 2017 and 18 September 2017.
2. *Half lubricant removed*: this condition concerns the monitoring tests of 21 September 2017 and 25 September 2017.
3. *No lubricant*: this condition concerns the tests from 2 October 2017 to 12 October 2017.

After the 13 Oct. 2017, the EMA reached a failure, given by a jam of the mechanical transmission. For a summary of the number of screw revolutions for each load and working condition, see Ref. [1]. Condition monitoring approaches, based on the data in Ref. [3], have been proposed in Refs. [1,[4], [5], [6]].

Summarizing, the following interventions were performed to accelerate the degradation process:

• we removed the anti-rotation device which is able to compensate small radial load components;
• we left three out of eight circuits with balls in the ballscrew;
• we set the radial load equal to 17% of axial load;
• We progressively remove lubricant in ballscrew/nut assembly.

A summary of the number of screw revolutions for each load and working condition is reported in Table 4, that shows how the majority of the tests were performed with no lubricant, in both endurance (800 N) and monitoring (300 N) tests.

**Table 4:** Number of screw revolutions after anti-rotation removal.

	Normal lubricant	Poor lubricant	No lubricant	Total
H0: 300 N	185.609	250.388	333.023	**769.020**
H1: 800 N	146.846	145.212	371.579	**663.637**
**Total**	**332.455**	**395.600**	**704.602**	**1.432.657**

The total revolutions for each load-lubricant condition are highlighted in bold text.

### 2.3 Access the measurements data inside the provided files.

The raw measurements are stored as private attributes of a specifically defined class. The provided files contain an object (called data_table) of such class, contains the raw measurements. In order to retrieve them, it is necessary to use the following syntax in MatLab:

variable = data_table.get(desired_variable, load, off, amp, freq);

where desired_variable, load, off, amp, and freq are char vectors that can assume the following values:

• desired_variable: indicates the raw physical variable to be retrieved. Its value is one of the “Stored variable names” values in Table 1
• load: indicates the test load. For monitoring test, the only possible value is ‘300 N’
• amps: indicates the amplitude of the reference profile. It can be one of {‘5mm’,‘10mm’};

In order to correctly import the data, it is necessary to first load the MatLab library that defines the objects’ class. To do this, download the files at the following link: https://github.com/CALUnibg/REPRISE_shared.git, and import in the MatLab path the folder “Import_data_phase1∖”.
